# Complete mitochondrial genome of the North American *Rhus* gall aphid *Melaphis rhois* (Hemiptera: Aphididae: Eriosomatinae)

**DOI:** 10.1080/23802359.2017.1303345

**Published:** 2017-03-23

**Authors:** Zhu-Mei Ren, Jun Wen

**Affiliations:** a School of Life Science, Shanxi University, Taiyuan, Shanxi, China;; b Department of Botany, MRC-166, National Museum of Natural History, Smithsonian Institution, Washington, DC, USA

**Keywords:** *Rhus* gall aphid, *Melaphis rhois*, Hemiptera, mitochondrial genome

## Abstract

We sequenced the complete mitochondrial genome (mitogenome) for the North American *Rhus* gall aphid species *Melaphis rhois*. The mitogenome is 15,436 bp in length with a high A + T content of 82.7%, consisting of 13 protein-coding genes, 22 tRNA genes, 2 rRNA genes, and a control region. Its gene order is identical to that of the eastern Asian species *Schlechtendalia chinensis*. All protein-coding genes start with a typical ATN codon and terminate with a TAA codon except *COI* and *ND4* by a single T residue. All the tRNAs except *tRNA^Cys^* formed a clover-leaf secondary structure. The mitogenome phylogeny of Aphididae suggests that *M. rhois* is most closely related to the eastern Asian *Rhus* gall aphid *S. chinensis* with the present sampling scheme.

The *Rhus* gall aphids belong to tribe Fordini (Hemiptera: Aphididae: Eriosomatinae) (Favret 2017) and were sometimes placed in subtribe Melaphidina (Remaudière & Remaudière 1997). Melaphidina aphids are primarily an Asian group with only one species *Melaphis rhois* in North America, exhibiting a biogeographic disjunction between eastern Asia and eastern North America (Wen 1999; Zhang et al. 1999; Ren et al. 2013). Up to now, only one complete mitochondrial genome (mitogenome) was reported in Eriosomatinae (Ren et al. 2016). We herein sequenced the complete mitochondrial genome of the unique North American *M. rhois* (GenBank accession KY624581).

All aphid individuals of a *Rhus* gall are of parthenogenetic generations. *Melaphis rhois* individuals from a gall were collected on *Rhus glabra* in Ohio (Columbus, 39**°**24^′^21.46^″^N, 84**°**23^′^37.20^″^W, altitude 238 m), USA (voucher deposited at the School of Life Science, Shanxi University, Taiyuan, China; Voucher no. A3037).

We obtained the mitogenome sequence of *M. rhois* using the shot-gun genome skimming method (Zimmer & Wen 2015) on an Illumina NextSeq 500 platform. The mitogenome sequence assembly used the eastern Asian species *Schlechtendalia chinensis* as the reference genome. We also did a *de novo* assembly with Velvet (Zerbino & Birney 2008).

The complete mitogenome of *M. rhois* is 15,436 bp in length, which contains 13 protein-coding genes (PCGs), 22 transfer RNA (tRNA) genes, 2 ribosomal RNA genes (*rrnL* and *rrnS*), and a control region. The gene order is the same as that of *Schlechtendalia chinensis.* All protein-coding genes have typical initiation and termination codons of ATN and TAA, respectively, except that *COI* and *ND4* each terminate with a single T. The 22 tRNA genes range from 63 bp to 73 bp, and all except *tRNA^Cys^* exhibit a classical clover-leaf secondary structure, which we predicted with tRNAscan-SE v1.21 and/or RNAstructure (Lowe & Eddy 1997; Bellaousov et al. 2013). The *tRNA^Cys^* was determined based on comparisons to other aphid mitogenomes. The mitogenome of *M. rhois* has a base composition of A (44.6%), T (38.1%), C (11.3%), G (6.0%), and an A + T content (82.7%). The *rrnL* gene is 1270 bp with an A + T content 84.7%, while the *rrnS* gene is 772 bp with an A + T content 83.6%. The control region spans 703 bp and is located between *rrnS* and *trnI* with an A + T content 85.0%. The repeat region unique to some Aphidinae species is present in the close relative *Schlechtendalia chinensis,* but absent in *M. rhois*.

We employed the maximum-likelihood method to estimate the phylogeny of Aphididae using 13 PCG genes and 2 rRNA genes (Figure 1). In this phylogeny, *M. rhois* is sister to *Schlechtendalia chinensis*. The mitogenome of *M. rhois*, the only North American species of the *Rhus* gall aphids, will be an important addition to explore the evolution of the biogeographic disjunction between eastern Asia and North America (Wen et al. 2016).

**Figure 1.**
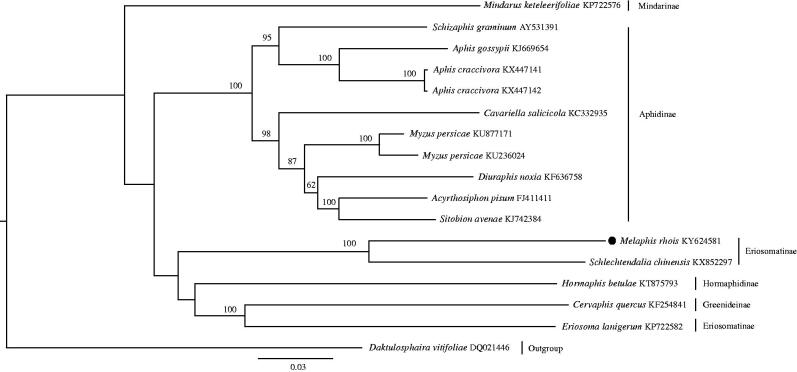
The maximum-likelihood tree of *M. rhois* and 16 accessions of Aphididae downloaded from GenBank using *Daktulosphaira vitifoliae* as an outgroup. Numbers above the branches indicate the bootstrap support values ≥50%. GenBank accession numbers and subfamily affiliations were indicated to the right of the terminals.
